# Correspondence: Atypical spinal infections in the era of multidrug resistance: knowledge gaps and a proposed management algorithm

**DOI:** 10.1097/MS9.0000000000004525

**Published:** 2025-12-12

**Authors:** Ahmad Furqan Anjum, Ali Rizwan Faisal, Hermann Yokolo

**Affiliations:** a Department of Orthopaedics, Shaikh Zayed Postgraduate Medical Institute (SZPGMI), Lahore, Pakistan; b Department of Research, Medical Research Circle (MedReC), Goma, DR Congo

*Dear Editor,*

Spinal infections pose a significant global health burden due to their association with high morbidity, neurological deficits, and the risk of structural instability. While pyogenic spinal infections benefit from standardized guidelines and established therapeutic strategies, atypical spinal infections (ASIs) particularly those caused by Mycobacterium tuberculosis and fungi remain under-recognized and poorly addressed. Pott’s disease, representing the most common form of skeletal tuberculosis, exemplifies the unique diagnostic and therapeutic challenges of ASIs. The growing prevalence of multidrug-resistant (MDR) and extensively drug-resistant (XDR) tuberculosis further complicates management and exposes critical gaps in current practice^[1]^. This article complies with the TITAN 2025 guidelines – repressing the reporting and use of AI^[2]^.

ASIs are often characterized by indolent progression and non-specific clinical features, which contribute to delayed recognition. Conventional culture techniques demonstrate poor sensitivity, particularly for tuberculosis and fungal pathogens, leading to missed or late diagnoses. Although molecular tools such as nucleic acid amplification tests (NAAT), GeneXpert, and metagenomic next-generation sequencing (mNGS) have shown high diagnostic accuracy, they remain underutilized in routine clinical practice^[3]^. A lack of standardized diagnostic algorithms integrating these modalities continues to hinder early detection and appropriate therapeutic decision-making.

Uncertainty persists regarding the optimal drug regimens and treatment duration for resistant forms of ASIs. In MDR-TB and resistant fungal infections, reliance on second-line agents is common, yet these regimens are often toxic, poorly tolerated, and variably effective^[4]^. This lack of consensus leads to heterogeneity in treatment practices, raising concerns about both efficacy and patient safety.

The role of surgery in ASIs remains ambiguous, with considerable variability in clinical decision-making across centers. Criteria for decompression, stabilization, or radical debridement are often dictated by individual surgeon judgment rather than evidence-based protocols. This lack of standardization is particularly concerning in cases complicated by neurological deficits, instability, or uncontrolled sepsis^[5]^. Developing clearer guidelines for surgical indications is essential to reduce practice variation and optimize outcomes.

Despite the increasing recognition of ASIs, multicenter registries and collaborative data-sharing platforms remain limited. The absence of robust real-world data restricts the development of evidence-based guidelines and hampers the evaluation of emerging diagnostic and therapeutic modalities^[6]^. Global collaborations are needed to generate outcome-driven evidence that can refine and standardize care pathways.

Addressing these gaps requires a multifaceted approach. First, diagnostic algorithms should integrate imaging, biopsy, and rapid molecular assays to enable timely recognition and initiation of treatment. Second, therapeutic protocols need to be adapted to the challenges of drug resistance, balancing efficacy with tolerability. Third, surgical guidelines must be standardized with defined criteria for intervention. Finally, establishing multicenter registries and fostering international collaborations will be critical to produce real-world outcome data and facilitate the continuous refinement of management strategies. A proposed diagnostic and management algorithm is illustrated in Fig. 1.

**Figure 1.**
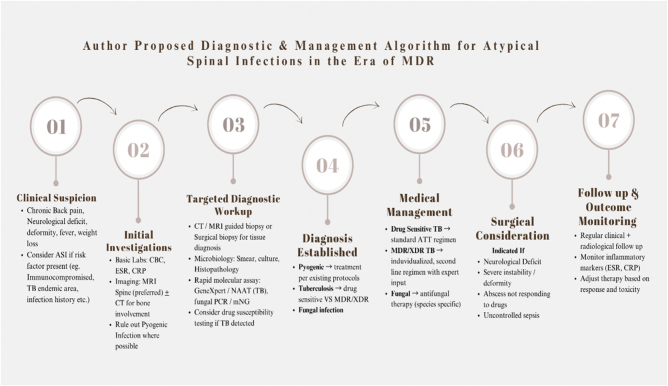
Proposed diagnostic and management algorithm for atypical spinal infections in the era of multidrug resistance. The algorithm emphasizes early suspicion, incorporation of rapid molecular diagnostics, individualized medical therapy, and clear surgical indications to address current knowledge gaps.

In Conclusion, Atypical spinal infections in the era of multidrug resistance remain a neglected frontier of spinal pathology. Bridging diagnostic, therapeutic, surgical, and research gaps through standardized guidelines and collaborative networks is essential to improve patient outcomes and reduce morbidity.

## Data Availability

Not applicable.
